# A Warning Outbreak

**Published:** 1891-12-12

**Authors:** 


### The Hospital

A Weekly Journal of

Science, Medicine, Nursing, and Philanthropy.

*For Hospitals, Asylums, Infirmaries, Dispensaries, Medical Practitioners, Students, Nurses, and the Charitable Public.*

Vol. XI.—No. 272.] [Dec. 12, 1891.

### A WARNING OUTBREAK.

There is a "Peace Society" in this country; and it devotes itself naturally to the modification and suppression of that temper in men which makes for war. There is also an Epidemiological Society, whose business it is to study disease epidemics, their true nature, their causes, and the means whereby they may be prevented. The great world takes very little notice of either of these societies: even learned persons pass them by as being fit only for specialists. Some day the world will know better, we trust. Persons of general learning will devote their trained minds to such eminently practical questions as epidemics that kill, and might be restrained from killing. "All that a man hath will he give for his life," said the devil. But that was in ancient and very primitive times. The majority of people to-day do not appear to care one straw for dangers that may lie in wait for their lives, unless those dangers are as evident to the senses as roaring lions, or as gross as putrefying heaps of slain on a battle-field.

This little preface is by way of chiding, for their own good, non-medical readers who leave the consideration of epidemic diseases entirely to specialists. There has just occurred a very peculiar and somewhat alarming outbreak at the Paddington Infirmary. All London would do well to turn its attention to this outbreak; because there is hardly any doubt that the same distressing and injurious affection which has seized upon 163 persons in a total population of 846 at Paddington may easily sieze upon a similar proportion of the whole of the inhabitants of London. The disease, though not very fatal, is one of the most unpleasant affections with which one can be attacked, and also one of the most prostrating. There are two features about it which appeal to the non-medical mind; first, it is a skin disease attacking many parts, and in some cases, the whole, of the body: what the doctors call "a universal dermatitis." Secondly, it usually lasts as long as seven or eight weeks. To read of such a disease makes the flesh of the sensitive man "creep" with disgust. But what must it be to be "laid up" with it for seven or eight weeks! It is as depressing as influenza, and ten times more disgusting, whilst it lasts three or four times as long.

Doctors, of course, are exercised about the pathology, the real nature of the affection; and with much reason. A "dermatitis," the physicians say the disease is; in plain English, an inflammation of the skin; and an extremely painful and distressing one. They do not seem to know whether to call it an eczema, or a pityriasis, or a herpes: and it really does not matter which of these names they give it; for pityriasis is a small cousin of eczema, and herpes is, at least, a connexion, if not a blood relation. In fact, this new disease, if it be a new one, may serve the useful purpose of making thc average practitioner think out the true nature and underlying causes of these three skin affections with a view to their more philosophic classification and still more to their effective treatment. For our part, we are quite satisfied that the disease is properly named a "dermatitis," and that it reveals in its peculiar characteristics a near kinship to eczema.

But that is only a part, though admittedly a fundamental part, of what we want to know about it. Why should nearly one in every four of the patients in the Paddington Infirmary have been seized by an eczematous dermatitis within the four months dating from the first of July to the thirty-first of October? This is the prime question to which we want an answer. Now it is pretty evident that the cause or causes must have been either in the patients themselves, or in their environment at the hospital, or in both. That it was not exclusively in the patients themselves is proved by the fact that not one of them entered the hospital with the disease upon him. Every one of them acquired it in the building. It would appear then to be certain that there was something in the circumstances of the institution which led to such a peculiar and distressing outbreak. What was it? Was it the food the patients ate? Or that they could not get to eat? Was it the water they drank? Was it the air they breathed? Was it the medicine which was administered to them? Or was it a host of invading microbes coming like the Vikings of old, we know not whence, and departing when they had done their deadly work, we know not where?

The microbe theory has one merit—it furnishes us with a solution that is at least easy. But is it the truth? Have we solid ground for feeling convinced that a casual army of microbes, like an invading host of locusts, swooped down upon the Paddington Infirmary early in July, and there remained until the end of October, and then took their departure? The thing is of course possible. There is also the possibility that the microbes were not foreigners at all, but indigenous to the soil; and that they had suddenly developed in overwhelming strength and numbers under certain favouring circumstances within the hospital which have not yet been discovered.

There are, however, other possible explanations, and it is one of these which, if it should prove to be the true one, very nearly concerns both the general public and its ordinary medical practitioners. We are persuaded that the nervous system plays a prominent part in much of the disease which prevails in large towns at the present time. The atmosphere of such towns is dense with fog and smoke, and is charged with impurities innumerable. It is, therefore, always depressing to the vitality of those who live in it, and sometimes actively poisonous. A depressed vitality results in an enfeebled nervous system; and an enfeebled nervous system is accompanied by poor digestion, imperfect elimination, weak muscles, and impaired activity of all kinds. What had the nervous systems of the 163 victims at Paddington to do with their selection by the disease? Much, probably. The affection itself showed certain well-marked characteristics, which in the most typical cases were almost uniformly present. First, the part of the skin affected became red; then small vesicles, or a running discharge followed: this was succeeded by a shedding of the outer skin in scales or flakes; and finally there remained as the result of these processes a thickened, hardened, and wrinkled condition of the portions of the skin affected. In many cases, what may be called the new skin presented a raw, parchment-like appearance, and was smooth and shiny, and sometimes cracked.

Now M. Charcot has shown that affections of the nerves and nerve centres produce certain characteristic phenomena in the skin, as well, of course, as in other parts of the body. Those skin phenomena are largely of the herpetic and eczematous order. They include vesicles and bullæ, eczema properly so called, erythema, with swelling and thickening of the skin, and also a smooth, shining, glossy condition, accompanied by portions that are cracked and wrinkled. The American physicians, Drs. Weir Mitchel, Morehouse, and Keen, had many opportunities, during the great war in the States, of observing the results, in the skin and elsewhere, of wounds and injuries to the nerves. The skin conditions resulting from wounds of the nerves were similar to those just described; that is to say, the skin became red, developed small vesicles, with larger bullæ or blebs, and, in some cases, true eczematous eruptions; it was often also smooth, shiny, and cracked at later stages.

Our contention is that an extremely depressed condition of the nervous system has had much to do with the outbreak of what is considered by some to have been a new disease at the Paddington Infirmary. Many of the circumstances are in favour of this conclusion. The patients belonged largely to the pauper class, and were, therefore, ill-fed and weakly before entering the hospital; they were probably not too well fed in the hospital; the older and feebler among the inmates suffered much more than the younger and stronger. Between the ages of thirty and forty only six per cent. were attacked, but between the ages of sixty and seventy thirty-eight per cent. Of all the officials in the institution, including the doctors and the nurses, who may be presumed to have been properly fed and in good health, only two were attacked, and those not severely. Moreover, treatment by stimulants seems to have been by far the most efficacious. The practical conclusion, therefore, is that since a depressed nervous system is eminently conducive to selection in all kinds of epidemics, every man and woman of real intelligence will spare neither pains nor expense in building up within himself a nervous system which is thoroughly robust.

